# YouTube as a Source of Influenza Vaccine Information in Spanish

**DOI:** 10.3390/ijerph18020727

**Published:** 2021-01-15

**Authors:** Ignacio Hernández-García, Teresa Giménez-Júlvez

**Affiliations:** 1 Department of Preventive Medicine and Public Health, Lozano Blesa University Clinical Hospital of Zaragoza, Calle San Juan Bosco 15, 50009 Zaragoza, Spain; 2 Health Services Research Group of Aragon (GRISSA), Aragon Institute for Health Research (IISA), Calle San Juan Bosco 15, 50009 Zaragoza, Spain; 3 Department of Preventive Medicine and Public Health, Miguel Servet University Hospital of Zaragoza, Paseo Isabel la Católica 1, 50009 Zaragoza, Spain; tgimenez@salud.aragon.es

**Keywords:** influenza vaccine, YouTube, Spanish, information, hoaxes, evaluation

## Abstract

Our objective was to analyze the information in Spanish on YouTube about the influenza vaccine. In August 2020, a search was conducted on YouTube using the terms “Vacuna gripe”, “Vacuna influenza”, and “Vacuna gripa”. Associations between the type of authorship, country of publication, and other variables (such as tone, hoaxes, and vaccination recommendations) were studied via univariate analysis. A total of 100 videos were evaluated; 57.0% were created in Mexico (24.0%), Argentina (17.0%), and Spain (16.0%), and 74.0% were produced by mass media or health professionals. Positive messages were detected in 65.0%. The main topics were the benefits of the vaccine (59.0%) and adverse effects (39.0%). Hoaxes were detected in 19 videos. User-generated content, compared to that of health professionals, showed a higher probability of hoaxes (odds ratio (OR) = 15.56), a lower positive tone (OR = 0.04), and less evidence of recommendations to vaccinate pregnant individuals (OR = 0.09) and people aged 60/65 or older. Videos published in Spain, in comparison with those from Hispanic America, presented significant differences in the positive tone of their messages (OR = 0.19) and in the evidence of the benefits of vaccination (OR = 0.32). A higher probability of hoaxes was detected in videos from Spain and the USA. Information in Spanish about the influenza vaccine on YouTube is usually not very complete. Spanish health professionals are urged to produce pro-vaccination videos that counteract hoaxes, and users in Hispanic America should be advised to consult videos produced in Hispanic American countries by health professionals to obtain reliable information.

## 1. Introduction

YouTube is the second biggest search engine and the second most visited website in the world [1]. Every day, YouTube exceeds 2 billion views, with the average user spending at least 15 min on the website [2]. Moreover, YouTube is an increasingly important source of health information and has the capacity to influence its users, e.g., regarding their vaccination habits [3]. For this reason, a strategy proposed by several scientific societies to increase influenza vaccination coverage is to use social networks to spread official indications and raise awareness of the importance of the vaccine [4].

However, the information shown on YouTube often lacks scientific rigor because anyone can upload such content [2]. For this reason, YouTube contains many videos that may be misleading [5].

Appropriate YouTube content can benefit health organizations by ensuring that the population properly implements measures to control the spread of infectious diseases; however, misleading videos can contribute to a failure to contain these diseases [6]. In particular, several authors have suggested that the spread of disinformation on the web may be associated with declines in vaccination coverage among the population [7,8]. For this reason, monitoring the information available in the media, such as YouTube, could be useful in assessing the level of vaccine hesitation and planning and conducting effective information campaigns [7].

In this context, infodemiological studies are necessary, since they can provide valuable insights into health-related behaviors of populations. Infodemiology is the science of distribution and determinants of information on the Internet or in a population, and it has the aim of informing public health and public policy [9,10]. Examples of infodemiology applications include the identification and monitoring of public-health-relevant publications on the Internet, measuring information diffusion, and analyzing how people search and navigate on the Internet for health-related information as well as how they communicate and share this information [9,10].

Multiple authors have carried out infodemiological studies that analyzed the characteristics of YouTube videos providing information about several vaccines [8,11,12,13,14,15,16,17]. However, information in Spanish about the influenza vaccine has been poorly studied because, among other things, scholars have not analyzed the incorrect information available on YouTube about this vaccine [18].

This research was carried out to analyze the information in Spanish on YouTube about the influenza vaccine. In particular, we addressed the following research questions (RQ):

- RQ 1: What are the general characteristics of the videos?
- RQ 2: What are the temporal distribution and the positive tone of the videos?
- RQ 3: What is the information related to the influenza vaccine discussed in the videos?
- RQ 4: What are the hoaxes and conspiracy theories related to the influenza vaccine discussed in the videos, and when and by whom were such videos published?

In addition, under the hypothesis that the variables type of authorship of the video and country of publication are related to certain characteristics of the videos, we addressed the following RQ:

- RQ 5: Is there any relationship between the type of authorship of the video and the year of publication, number of views/likes/dislikes/comments, duration, type of publication, positive tone of the message, and information discussed regarding the influenza vaccine?
- RQ 6: Is there any relationship between the country of publication of the video and the year of publication, number of views/likes/dislikes/comments, duration, type of publication, positive tone of the message, and information discussed regarding the influenza vaccine?

## 2. Materials and Methods

On 26 August 2020, a cross-sectional study of the data was conducted by entering the terms “Vacuna gripe”, “Vacuna influenza”, and “Vacuna gripa” into the YouTube search engine. The videos were sorted according to the number of their views. After applying the inclusion criteria (published since 1 January 2015) and the exclusion criteria (not available for viewing, not providing information on the influenza vaccine, language other than Spanish, and duplicated video), we selected the 100 most viewed videos. We decided to limit our analysis to the 100 most viewed videos since this is a common and accepted procedure when investigating YouTube videos [19,20,21].

The full name of the video, its URL (Uniform Resource Locator), and the information corresponding to the following variables were recorded: date of publication, country of publication, number of views, number of likes, number of dislikes, number of comments, duration, type of publication, and type of authorship (representing the person or organization that produced the video, classified into 4 categories: “mass media”, including television and journals; “health professionals”, including healthcare workers, medical centers, and official public health organizations; “user-generated content”, a lay person’s opinion about the issue; and “others”, videos that did not belong to any other category [3,22]).

The videos were categorized according to message tone, adopting a classification used previously in other studies [3,14,15,23]: “positive”, vaccination is clearly recommended, i.e., the central message of the video supports vaccination, portraying it positively (e.g., described the benefits and safety of the influenza vaccine, described vaccination as a social good, or encouraged people to receive such vaccine); “negative”, arguments are put forward against vaccination, i.e., the main message portrays vaccination negatively (e.g., emphasized the risk of vaccination, advocated against vaccination, promoted distrust in vaccine science, made allegations of conspiracy or collusion between supporters of vaccination and manufacturers); “ambiguous”, information is given for and against the vaccine, i.e., a beneficial or social good is countered by negative experiences; and “neutral”, there are no statements related to either approval or disapproval of the vaccine. Moreover, we recorded whether the videos provided information on the benefits, adverse effects, costs, dosage, and influenza vaccination recommendations for persons 60/65 years of age or older; pregnant women; healthcare workers; children; persons with chronic cardiovascular diseases; and those with chronic lung diseases, diabetes mellitus, obesity, cancer, infection by human immunodeficiency virus, and chronic kidney disease. Hoaxes and conspiracy theories related to the influenza vaccine were also recorded.

For assessing the message tone, 2 authors (I.H.-G. and T.G.-J.) independently viewed the selected videos and determined the tone of their message, according to the previously described definitions of “positive”, “negative”, “ambiguous”, or “neutral” tone. Both authors had previous experience in assessing the tone of the YouTube videos message in relation to vaccines [14]. A concordance analysis was performed between the two using the Kappa index, without obtaining any discrepancies (kappa = 1.0).

A descriptive analysis of the variables was performed; quantitative variables were expressed as the median (range), while qualitative variables were expressed as the absolute (*n*) and relative (%) frequencies. The associations between the type of authorship (categorized in mass media, user-generated content, health professionals, and others) and the year of publication, type of publication, tone of the message (categorized in positive and other), and information discussed regarding the influenza vaccine were studied. For this purpose, a chi-squared test or Fisher’s exact test were used. The magnitude of the associations was quantified with the odds ratio (OR), and a 95% confidence interval (CI) was obtained with a univariate logistic regression analysis. The associations between the type of authorship (categorized in mass media, user-generated content, health professionals, and others) and the video duration, number of views, number of likes, number of dislikes, and number of comments were studied. After using the Kolmogorov–Smirnov test to ensure that no variables followed a normal distribution, we compared the median values with a Mann–Whitney *U*-test.

The associations between the country of publication (categorized in Spain, the United States of America, Hispanic American countries, and others/unknown) and the year of publication, type of publication, tone of the message (categorized in positive and other), and information discussed regarding the influenza vaccine were studied. For this purpose, a chi-squared test or Fisher’s exact test were used. The magnitude of the associations was quantified with the odds ratio (OR) and a 95% confidence interval (CI) was obtained with a univariate logistic regression analysis. The associations between the country of publication (categorized in Spain, the United States of America, Hispanic American countries, and others/unknown) and the video duration, number of views, number of likes, number of dislikes, and number of comments were studied. For this purpose, the median values were compared with a Mann–Whitney *U*-test. Moreover, a chi-squared test or Fisher’s exact test were used to study the association between the year of publication of the video and the positive tone of the message, as well as the detection of hoaxes/conspiracy theories.

The level of statistical significance for comparison of the hypotheses was established at *p* < 0.05. All the statistical analyses were performed using SPSS v25 and EpiInfo.

## 3. Results

The 100 most widely viewed videos, published between 1 January 2015 and 26 August 2020, were analyzed. There were 55 videos excluded and replaced: 4 due to irrelevance (not about the influenza vaccine), 6 that were not available for viewing, and 45 as triplicated videos.

### 3.1. RQ 1: What Are the General Characteristics of the Videos?

Of the 100 videos, the majority (*n* = 70, 70.0%) were produced in Mexico (24.0%), Argentina (17.0%), Spain (16.0%), and Chile (13.0%) (Table 1). The median length (range) was 187.5 s (10–4769), and the number of views, likes, dislikes and comments were, respectively, 10,553.5 (2936–294,969), 69.5 (0–10,089), 11.5 (0–546), and 16.5 (0–2072). With regard to the type of authorship and publication, 74.0% of the videos were produced by mass media or health professionals, and 70.0% of the videos were news pieces or material created by a user (Table 1).

### 3.2. RQ 2: What Are the Temporal Distribution and the Positive Tone of the Videos?

Among the most widely viewed YouTube videos, almost half (*n* = 48; 48.0%) were published in the years 2019 and 2020 (Table 2). In total, 65.0% of the videos provided positive messages regarding the use of the vaccine, while negative, neutral, or ambiguous messages were in 16.0%, 15.0%, and 4.0% of the videos, respectively.

Among the 65 videos with a positive tone, 4 (6.2%) were published in 2015, 8 (12.3%) in 2016, 7 (10.8%) in 2017, 11 (16.9%) in 2018, 15 (23.0%) in 2019, and 20 (30.8%) in 2020 (*p* > 0.05).

### 3.3. RQ 3: What Is the Information Related to the Influenza Vaccine Discussed in the Videos?

The most frequent information in the videos was about the benefits of the vaccine (59.0%), adverse effects (39.0%), recommendations for any trimester of pregnancy (36.0%), and recommendations for people aged 65 years or older (27.0%) (Table 3).

### 3.4. RQ 4: What Are the Hoaxes and Conspiracy Theories Related to the Influenza Vaccine Discussed in the Videos, and When and by Whom Were Such Videos Published?

Hoaxes and conspiracy theories were detected in 19 videos (19.0%) (14 corresponded to user-generated content, 3 videos were produced by health professionals, and 2 were released by mass media). In particular, 12 videos indicated that the influenza vaccine is not safe, because, among other factors, it is formed by formaldehyde and thiomerosal (derived from mercury), which are neurotoxic and have harmful effects on people’s health, or because influenza vaccination is associated with autism, Alzheimer’s disease, infant mortality, autoimmune diseases, or the cause of coronavirus disease 2019 (COVID-19). Moreover, seven videos indicated that the influenza vaccine is totally ineffective and constitutes a scam by the big pharmaceutical industries to make money from vulnerable people. Three videos specified that it is better to drink certain infusions to avoid influenza than to get vaccinated; two videos indicated that the influenza vaccine is used to control our minds; and two videos explained that influenza vaccines spread influenza (Table 4).

The three videos produced by health professionals who perpetuated hoaxes dealt with the following topics: the influenza vaccine can produce influenza; the vaccine is contraindicated in pregnant women, those with chronic degenerative or chronic infectious diseases, or those who have some type of immunosuppression; and the influenza vaccine causes fibromyalgia and multiple sclerosis.

Among the 19 videos with hoaxes and conspiracy theories, 2 (10.5%) were published in 2015, 3 (15.8%) in 2016, 3 (15.8%) in 2017, 6 (31.6%) in 2018, 2 (10.5%) in 2019, and 3 (15.8%) in 2020 (*p* > 0.05).

### 3.5. RQ 5: Is There Any Relationship between the Type of Authorship of the Video and the Year of Publication, Number of Views/Likes/Dislikes/Comments, Duration, Type of Publication, Positive Tone of the Message, and Information Discussed Regarding the Influenza Vaccine?

According to the type of authorship, in the univariate analysis, the user-generated content, compared to the videos produced by health professionals, presented significant differences in their number of likes (a median (range) of 319.5 (0–3911) for the user-generated content and of 52 (3–1074) in those produced by health professionals (*p* = 0.006)); number of comments (median (range) of 34 (0–519) for user-generated contents and 3 (0–347) in those produced by health professionals (*p* = 0.031)); positive tone of the message (OR: 0.04 (95% CI: 0.01–0.19)) (Table 5); the presence of hoaxes (OR: 15.56 (95% CI: 3.32–72.93)); evidence of benefits from the vaccine (OR: 0.06 (95% CI: 0.01–0.29)); and recommendations for pregnant women (OR: 0.09 (95% CI: 0.02–0.46)), children, and persons aged 60/65 years or older (Table 6). There were also differences in the type of authorship and the frequency with which the video provided information on the recommendation to vaccinate all persons older than 6 months (Table 6).

In addition, videos produced by health professionals, compared to those of the mass media, showed significant differences in their duration (median (range), with 105 (10–1901) seconds in those produced by health professionals and 198 (35–4665) seconds in those of the mass media (*p* = 0.034)). No other significant differences were detected (*p* > 0.05).

### 3.6. RQ 6: Is There Any Relationship between the Country of Publication of the Video and the Year of Publication, Number of Views/Likes/Dislikes/Comments, Duration, Type of Publication, Positive Tone of the Message, and Information Discussed Regarding the Influenza Vaccine?

According to the country of publication, videos published in Spain, in comparison to those from Hispanic America, presented significant differences in the positive tone of their message (OR: 0.19 (95% CI: 0.06–0.61)) (Table 7), evidence for the benefits of vaccination (OR: 0.32 (95% CI: 0.10–0.99)), and information on recommendations to vaccinate children. In addition, significant differences were observed when comparing videos published from the United States of America (USA) with those from Hispanic America regarding the consistency of their recommendation to vaccinate children (OR: 0.11 (95% CI: 0.01–0.88)), people aged 60/65 or older (OR: 0.1 (95% CI: 0.01–0.83)), and people with chronic cardiovascular disease. A higher number of hoaxes was detected in videos from Spain and the USA compared to those from Hispanic American countries (Table 8). No other significant associations were found (*p* > 0.05).

## 4. Discussion

This study is the first to analyze the characteristics of YouTube videos that provide information in Spanish about the influenza vaccine by considering the three terms used in Spanish-speaking countries to refer to this virus (gripe, influenza, and gripa). In this way, we obtained results with greater validity at an international level than the only other published research of this type (to our knowledge), in which only videos obtained using the term “vacuna gripe” were evaluated [18].

The majority of the videos (65.0%) had a positive tone toward the vaccine’s use, which contrasts with the results of Yiannakoulias et al. [11], who evaluated 141 videos in English about the vaccine and observed that only 16.3% were in favor of it. This disparity according to language coincides with studies on other vaccines, such as those for human papillomavirus, in which a mostly positive tone was detected in more than three-quarters of the videos in Spanish [24], while in English, positions against the vaccine’s use were the most frequent (51.7%) [15].

The median number of views detected (10,553.5) was also much higher than those found by Yiannakoulias et al. (67.5) [11]. This may be because this author analyzed a sample of selected videos in the order in which they appeared in the YouTube search results. In addition, since 36.0% of the videos corresponded to news previously broadcast by mass media, they were potentially seen by many more people than those who accessed the version on YouTube.

In just over half of the videos (51.0%), the type of authorship corresponded to mass media, which is consistent with the results found for Spanish videos on the Bexsero vaccine (45.2%) [25] but differs from the results of Tuells et al., who observed that only 14.1% of the Spanish videos on human papillomavirus vaccines were produced by mass media [24].

Although the main information provided corresponded to the benefits of the vaccine, a fact recognized worldwide [26], in 41.0% of the videos, a positive tone was not found, which indicates that the information in YouTube videos regarding the flu vaccine is often incomplete. This deficiency of information in YouTube videos that focus on the influenza vaccine can be confirmed by observing the low frequency of certain recommendations of the World Health Organization (WHO) [27] adopted in Spain [28], the USA [29], and Hispanic American countries [30,31], such as recommendations to vaccinate healthcare workers annually to reduce the risk of acquiring the influenza and transmitting it to their patients [28,29,32]. Similarly, other WHO recommendations adopted in such countries [27,28,29,30,31], such as vaccinating people over 65 years of age, did not appear in half of the videos, even if the recommendation is described as such, or in the context of expanding vaccination in certain countries by including a recommendation to vaccinate people over 60 years of age [30,31] or the entire population over 6 months of age [29]. Something similar was observed with the recommendation to vaccinate during any trimester of pregnancy, which did not appear in half of the videos, and the recommendation to vaccinate pregnant women only from the second trimester in certain countries [33]. For all these reasons, influenza vaccination coverage in Spanish-speaking countries can be improved [31,34,35]. Indeed, among other factors, vaccination coverage among healthcare workers is usually less than 46% [31,35], less than 55% in those over 60/65 years olf [31,34,35], and less than 41% in pregnant women [31,35]. Moreover, the information available on YouTube can influence the vaccination habits of its users [3]. Therefore, a possible measure to improve such coverage could be to use YouTube to officially disseminate these recommendations. Indeed, several scientific societies, such as the Spanish Association of Vaccinology and the Spanish Society of Preventive Medicine and Public Health, have proposed to use social networks to disseminate such indications and raise awareness of the importance of this vaccine as a strategy to increase influenza vaccination coverage [4]. The implementation and evaluation of the effectiveness of this measure could be the subject of future research.

The differences detected in the tone of the message, according to type of authorship, are congruent with other studies that have analyzed the information on YouTube about vaccines in general, in which the videos of health professionals have a more positive tone [3]. Likewise, the fact that these videos are more likely to provide reliable information confirms what other authors have described [13].

The finding that videos made by health professionals, compared to user-generated content, produce less interaction between YouTube users in terms of likes could be explained by the phenomenon called confirmation bias, which leads people to favor information that confirms their beliefs [36]. These behaviors create what is called an “echo chamber” in the new media [37], and our results confirm this feature.

On the other hand, the fact that videos published in Hispanic America were found to be more likely to provide certain vaccination recommendations, as well as a lower number of hoaxes, justifies promoting, among users residing in Hispanic American countries, the consultation of videos specifically produced in such countries by health professionals when they search on YouTube for information in Spanish about the influenza vaccine. Likewise, Spanish health professionals should be urged to publish information about this vaccine on YouTube, alluding to its benefits, counteracting the hoaxes, and making videos with a pro-vaccination tone to help change the tendency where videos produced in Spain less frequently have a positive tone, describe the benefits of the vaccine with less frequency, and provide a greater number of hoaxes than those in Hispanic American countries.

Hoaxes, or conspiracy theories, were detected in 19.0% of the videos. This represents a very worrying and novel finding (since previous research on the information available on YouTube about the influenza vaccine had not evaluated this aspect [11,18]). The dissemination of misinformation can cause fear and ultimately diminish the application of preventive measures [38,39], mainly among non-medically educated users for whom it is difficult to judge the reliability of health information online [38]. Perhaps this reason is why the WHO website provides information to refute the following five myths: “influenza is not serious so I don’t need the vaccine”; “the influenza vaccine can give me the flu”; “the influenza vaccine can cause severe side effects”; “I had the vaccine and still got the flu, so it doesn’t work”; and “I am pregnant so I shouldn’t get the influenza vaccine” [40].

However, on the basis of our findings, there is an urgent need for the WHO to counteract many more hoaxes. In addition, our findings could be used to guide the implementation of educational campaigns to correct misinformation about influenza vaccine in Spanish. In any case, to combat misinformation in YouTube, more strategies must be adopted, such as increasing editorial control within the platform [41], monitoring the spread of misinformation about vaccinations on YouTube, and encouraging governmental/academic organizations and new media to work together to fight false beliefs about vaccines [8].

The methodology used in this study is similar to that used by other authors [3,8,11,13,15,16,19,20,21,24,41], and the limitations are those intrinsic to the Internet. In this type of infodemiological research [9,10], the information available at any given time is analyzed [3,8,11,13,14,15,16,18,19,20,21,24,41]; however, the information online is constantly changing. In addition, the search terms were chosen by the authors under the assumption that a Spanish-speaking user would use one of them to perform simple searches on YouTube regarding influenza vaccine. The number of videos evaluated, although somewhat smaller than that used by some other authors (142) [16], was the same as, or greater than, that used in most studies of this type [13,14,19,20,21], in which 100 videos or less were included. In any case, our sample was able to obtain significant results.

## 5. Conclusions

This study shows that information on YouTube about the influenza vaccine is usually not very complete and that it differs according to the type of authorship and country of publication. Our findings suggest that Spanish health professionals should be more engaged in establishing a YouTube presence in order to provide reliable information, publishing pro-vaccination videos to respond to questions of the public about influenza vaccine and its hoaxes/conspiracy theories. Moreover, people residing in Hispanic America should be advised that, when looking for information related to this vaccine on YouTube, they should consult videos produced in Hispanic American countries by health professionals to obtain reliable information. These actions could be useful to allow Spanish-speaking citizens to make informed decisions about influenza vaccine so as to comply with vaccination recommendations.

## Figures and Tables

**Table 1:** General characteristics of the 100 videos.

Characteristics	Frequency, *n* (%)
**Country of publication**	
Mexico	24 (24.0)
Argentina	17 (17.0)
Spain	16 (16.0)
Chile	13 (13.0)
The United States of America	11 (11.0)
Colombia	3 (3.0)
Peru	3 (3.0)
Nicaragua	2 (2.0)
Costa Rica	2 (2.0)
France	2 (2.0)
Paraguay	1 (1.0)
Panama	1 (1.0)
Canada	1 (1.0)
Russia	1 (1.0)
Unknown	3 (3.0)
**Type of authorship**	
Mass media	51 (51.0)
Health professionals	23 (23.0)
User-generated content	20 (20.0)
Others	6 (6.0)
**Type of publication**	
News	36 (36.0)
Material created by the user	34 (34.0)
Interviews	18 (18.0)
Advertisements	8 (8.0)
Conferences	2 (2.0)
Documentaries	2 (2.0)

**Table 2:** Year of publication of the 100 videos.

Year of Publication	Frequency, *n* (%)
2015	9 (9.0)
2016	11 (11.0)
2017	14 (14.0)
2018	18 (18.0)
2019	20 (20.0)
2020	28 (28.0)

**Table 3:** Information related to the influenza vaccine discussed in the videos.

Information Related to the Influenza Vaccine	Discussed, *n* (%)	Not Discussed, *n* (%)
Benefits of the vaccine	59 (59.0)	41 (41.0)
Adverse effects of the vaccine	39 (39.0)	61 (61.0)
Recommendations for any trimester of pregnancy	36 (36.0)	64 (64.0)
Recommendations for people aged 65 years of age or older	27 (27.0)	73 (73.0)
Recommendations for people with chronic lung diseases	26 (26.0)	74 (74.0)
Recommendations for people with diabetes mellitus	21 (21.0)	79 (79.0)
Recommendations for people with chronic cardiovascular diseases	20 (20.0)	80 (80.0)
Hoaxes and conspiracy theories	19 (19.0)	81 (81.9)
Recommendations for healthcare workers	18 (18.0)	82 (82.0)
Costs of the vaccine	18 (18.0)	82 (82.0)
Recommendations for children of 6 months to 5 years	17 (17.0)	83 (83.0)
Recommendations for people with cancer	14 (14.0)	86 (86.0)
Description of the dosage	14 (14.0)	86 (86.0)
Recommendations for people with obesity	13 (13.0)	87 (87.0)
Recommendations for people of 60 or more years	11 (11.0)	89 (89.0)
Recommendations for all people older than 6 months	11 (11.0)	89 (89.0)
Recommendations for children aged 6 months to 2 years	10 (10.0)	90 (90.0)
Recommendations for people with HIV ^a^	9 (9.0)	91 (91.0)
Recommendations for people with chronic kidney disease	7 (7.0)	93 (93.0)
Recommendations for pregnant women from 13 weeks of gestation	5 (5.0)	95 (95.0)
Recommendations for children aged 6 months to 6 years	3 (3.0)	97 (97.0)
Recommendations for children aged 6 months to 10 years	3 (3.0)	97 (97.0)

^a^ HIV: human immunodeficiency virus.

**Table 4:** Hoaxes and conspiracy theories detected in the videos.

- The influenza vaccine is totally ineffective. - The influenza vaccine is a scam by the big pharmaceutical industries to make money from vulnerable people. - Influenza vaccines are used to control our minds—the vaccine is a way to introduce control agents/nanotechnology microchips into our bodies, in addition to experimental pathogens. - Influenza vaccines often make the flu worse. - It is better to drink celery, ginger, lemon, and pineapple juice than obtain an influenza shot. - It is best to drink hot tea made from lemons, oranges, pineapple, ginger, apple cider vinegar, turmeric, cayenne pepper, stevia or organic honey, and garlic than obtain an influenza shot.
- Drinking cod liver oil, thyme, turmeric or ginger tea, honey, and calendrus is a good alternative to the influenza vaccine. - Influenza vaccines are not safe. - Influenza vaccines spread the flu—people who receive influenza vaccines release 630 percent more influenza virus particles into the air compared to unvaccinated people. - The vaccine is toxic to health because it is made from formaldehyde and thiomerosal (derived from mercury), which are neurotoxins with harmful effects on people’s health. - The mercury and aluminum contained in influenza vaccines alter the immune system, causing it to not work properly and slowing it down. - Influenza vaccination among children is associated with autistic children.
- It has been shown that influenza vaccination is associated with Alzheimer’s disease. - Coronavirus disease 2019 (COVID-19) was produced by the influenza vaccine because it contains coronavirus. - Every year, thousands of people die from the influenza vaccine (without allergic reactions justifying all those deaths). - The influenza vaccine most likely killed United States Senator Jose Peralta by causing him to go into septic shock. - The increased use of influenza vaccines is directly related to an increase in all types of autoimmune diseases. - There is a correlation between increased rates of childhood influenza vaccination and increased infant mortality rates. - Influenza vaccination is harmful to a mother’s fetus and is linked to miscarriage. - Influenza vaccines can lead to influenza. - The influenza vaccine is contraindicated in people with chronic or degenerative diseases or those with some type of immunosuppression. - Influenza vaccines cause fibromyalgia. - Influenza vaccines cause multiple sclerosis.

**Table 5:** Type of authorship and tone of the message.

Tone	Type of Authorship	Available, *n* (%)	Unavailable, *n* (%)	OR (95% CI) ^a^	*p*
Positive tone of the message	Mass media	36 (55.4)	15 (42.9)	0.36 (0.09–1.39)	0.155
	User-generated content	4 (6.2)	16 (45.7)	0.04 (0.01–0.19)	0.000
	Others	5 (7.7)	1 (2.9)	0.75 (0.06–8.83)	1.000
	Health professionals	20 (30.7)	3 (8.5)	1	

^a^ OR (95% CI): odds ratio (95% confidence interval).

**Table 6:** Type of authorship and information discussed regarding the influenza vaccine.

Information Regarding Influenza Vaccine	Type of Authorship	Available, *n* (%)	Unavailable, *n* (%)	OR (95% CI) ^a^	*p*
Benefits of the vaccine	Mass media	35 (59.3)	16 (39.1)	0.77 (0.26–2.33)	0.648
	User-generated content	3 (5.1)	17 (41.5)	0.06 (0.01–0.29)	0.000
	Others	4 (6.8)	2 (4.9)	0.71 (0.10–4.89)	1.000
	Health professionals	17 (28.8)	6 (14.6)	1	
Adverse effects of the vaccine	Mass media	17 (43.6)	34 (55.7)	0.78 (0.28–2.16)	0.631
	User-generated content	13 (33.3)	7 (11.5)	2.89 (0.83–10.02)	0.094
	Others	0 (0)	6 (9.8)	-	0.138
	Health professionals	9 (23.1)	14 (23.0)	1	
Recommendations for pregnancy ^b^	Mass media	23 (56.1)	28 (47.5)	0.63 (0.23–1.70)	0.366
	User-generated content	2 (4.9)	18 (30.5)	0.09 (0.02–0.46)	0.002
	Others	3 (7.3)	3 (5.1)	0.77 (0.13–4.66)	1.000
	Health professionals	13 (31.7)	10 (16.9)	1	
Recommendations for children ^c^	Mass media	19 (57.6)	32 (47.8)	0.54 (0.20–1.47)	0.232
	User-generated content	0 (0)	20 (29.9)	-	0.000
	Others	2 (6.0)	4 (6.0)	0.46 (0.07–3.02)	0.651
	Health professionals	12 (36.4)	11 (16.4)	1	
Recommendations for people aged 60/65 years or older	Mass media	19 (50.0)	32 (51.6)	0.46 (0.17–1.24)	0.124
	User-generated content	2 (5.3)	18 (29.0)	-	0.002
	Others	4 (10.5)	2 (3.2)	1.54 (0.23–10.15)	1.000
	Health professionals	13 (34.2)	10 (16.1)	1	
Recommendations for people with chronic lung diseases	Mass media	11 (52.4)	40 (50.6)	0.99 (0.29–3.27)	0.987
	User-generated content	2 (9.5)	18 (22.8)	0.4 (0.07–2.34)	0.421
	Others	3 (14.3)	3 (3.8)	3.6 (0.55–23.65)	0.305
	Health professionals	5 (23.8)	18 (22.8)	1	
Recommendations for people with chronic cardiovascular disease	Mass media	12 (60.0)	39 (48.8)	1.46 (0.42–5.14)	0.762
	User-generated content	2 (10.0)	18 (22.5)	0.53 (0.09–3.24)	0.669
	Others	2 (10.0)	4 (5.0)	2.38 (0.32–17.74)	0.575
	Health professionals	4 (20.0)	19 (23.8)	1	
Recommendations for people with cancer	Mass media	9 (64.3)	42 (48.8)	1.43 (0.35–5.86)	0.743
	User-generated content	1 (7.1)	19 (22.1)	0.35 (0.04–3.67)	0.611
	Others	1 (7.1)	5 (5.8)	1.33 (0.11–15.71)	1.000
	Health professionals	3 (21.4)	20 (23.3)	1	
Recommendations for people with obesity	Mass media	9 (69.2)	42 (48.3)	1.43 (0.35–5.86)	0.743
	User-generated content	0 (0)	20 (23.0)	-	0.236
	Others	1 (7.7)	5 (5.7)	1.33 (0.11–15.71)	1.000
	Health professionals	3 (23.1)	20 (23.0)	1	
Recommendations for healthcare workers	Mass media	12 (66.7)	39 (47.5)	1.46 (0.42–5.14)	0.762
	User-generated content	0 (0)	20 (24.4)	-	0.111
	Others	2 (11.1)	4 (4.9)	2.38 (0.32–17.74)	0.575
	Health professionals	4 (22.2)	19 (23.2)	1	
Recommendations for people with HIV ^d^	Mass media	7 (77.8)	44 (48.4)	3.5 (0.41–30.26)	0.422
	User-generated content	0 (0)	20 (22.0)	-	1.000
	Others	1 (11.1)	5 (5.5)	4.4 (0.23–82.98)	0.377
	Health professionals	1 (11.1)	22 (24.1)	1	
Recommendations for people with chronic kidney disease	Mass media	5 (71.4)	46 (49.5)	2.39 (0.26–21.72)	0.659
	User-generated content	1 (14.3)	19 (20.4)	1.16 (0.07–19.79)	1.000
	Others	0 (0)	6 (6.5)	-	1.000
	Health professionals	1 (14.3)	22 (23.7)	1	
Recommendations for people with diabetes mellitus	Mass media	13 (61.8)	38 (48.1)	1.63 (0.47–5.66)	0.558
	User-generated content	2 (9.5)	18 (22.8)	0.53 (0.09–3.24)	0.669
	Others	2 (9.5)	4 (5.1)	2.38 (0.32–17.74)	0.575
	Health professionals	4 (19.1)	19 (24.0)	1	
Recommendations for all people older than 6 months	Mass media	3 (27.3)	48 (53.9)	0.14 (0.03–0.62)	0.008
	User-generated content	0 (0)	20 (22.5)	-	0.010
	Others	1 (9.1)	5 (5.6)	0.46 (0.05–4.67)	0.647
	Health professionals	7 (63.6)	16 (18.0)	1	
Costs of the vaccine	Mass media	11 (61.0)	40 (48.8)	0.99 (0.29–3.27)	0.987
	User-generated content	1 (5.6)	19 (23.2)	0.19 (0.02–1.78)	0.192
	Others	1 (5.6)	5 (6.1)	0.72 (0.07–7.66)	1.000
	Health professionals	5 (27.8)	18 (21.9)	1	
Description of the dosage	Mass media	9 (64.3)	42 (48.8)	0.77 (0.23–2.63)	0.679
	User-generated content	0 (0)	20 (23.3)	-	0.051
	Others	0 (0)	6 (7.0)	-	0.553
	Health professionals	5 (35.7)	18 (20.9)	1	
Hoaxes and conspiracy theories	Mass media	2 (10.5)	49 (60.5)	0.27 (0.04–1.75)	0.170
	User-generated content	14 (73.7)	6 (7.4)	15.56 (3.32–72.93)	0.000
	Others	0 (0)	6 (7.4)	-	1.000
	Health professionals	3 (15.8)	20 (24.7)	1	

^a^ OR (95% CI): odds ratio (95% confidence interval). ^b^ Recommendations for pregnancy (from 13 weeks of gestation or in any trimester of pregnancy). ^c^ Recommendations for children of 6 months to 2/5/6/10 years. ^d^ HIV: human immunodeficiency virus.

**Table 7:** Country of publication and tone of the message.

Tone	Country of Publication	Available, *n* (%)	Unavailable, *n* (%)	OR (95% CI) ^a^	*p*
Positive tone of the message	Spain	6 (9.2)	10 (28.6)	0.19 (0.06–0.61)	0.003
	United States of America	7 (10.8)	4 (11.4)	0.56 (0.15–2.16)	0.399
	Others/Unknown	2 (3.1)	5 (14.3)	0.13 (0.02–0.73)	0.009
	Hispanic American countries	50 (76.9)	16 (45.7)	1	

^a^ OR (95% CI): odds ratio (95% confidence interval).

**Table 8:** Country of publication and information discussed regarding the influenza vaccine.

Information Regarding Influenza Vaccine	Country of Publication	Available, *n* (%)	Unavailable, *n* (%)	OR (95% CI) ^a^	*p*
Benefits of the vaccine	Spain	6 (10.2)	10 (24.4)	0.32 (0.10–0.99)	0.044
	United States of America	8 (13.6)	3 (7.3)	1.43 (0.35–5.90)	0.625
	Others/Unknown	2 (3.4)	5 (12.2)	0.21 (0.04–1.19)	0.060
	Hispanic American countries	43 (72.9)	23 (56.1)	1	
Adverse effects of the vaccine	Spain	8 (20.5)	8 (13.1)	1.75 (0.58–5.26)	0.319
	United States of America	5 (12.8)	6 (9.8)	1.46 (0.40–5.29)	0.567
	Others/Unknown	2 (5.1)	5 (8.2)	0.7 (0.13–3.89)	0.684
	Hispanic American countries	24 (61.5)	42 (68.9)	1	
Recommendations for pregnancy ^b^	Spain	4 (9.8)	12 (20.3)	0.33 (0.09–1.14)	0.073
	United States of America	4 (9.8)	7 (11.9)	0.57 (0.15–2.14)	0.405
	Others/Unknown	0 (0)	7 (11.9)	-	0.012
	Hispanic American countries	33 (80.5)	33 (55.9)	1	
Recommendations for children ^c^	Spain	0 (0)	16 (23.9)	-	0.000
	United States of America	1 (3.0)	10 (14.9)	0.11 (0.01–0.88)	0.015
	Others/Unknown	0 (0)	7 (10.4)	-	0.015
	Hispanic American countries	32 (97.0)	34 (50.7)	1	
Recommendations for people aged 60/65 or older	Spain	4 (10.5)	12 (19.4)	0.33 (0.09–1.14)	0.073
	United States of America	1 (2.6)	10 (16.1)	0.1 (0.01–0.83)	0.012
	Others/Unknown	0 (0)	7 (11.3)	-	0.012
	Hispanic American countries	33 (86.8)	33 (53.2)	1	
Recommendations for people with chronic lung diseases	Spain	2 (9.5)	14 (17.7)	0.38 (0.08–1.85)	0.219
	United States of America	1 (4.8)	10 (12.7)	0.27 (0.03–2.24)	0.198
	Others/Unknown	0 (0)	7 (8.9)	-	0.114
	Hispanic American countries	18 (85.7)	48 (60.8)	1	
Recommendations for people with chronic cardiovascular disease	Spain	2 (10.0)	14 (17.5)	0.38 (0.08–1.85)	0.219
	United States of America	0 (0)	11 (13.8)	-	0.049
	Others/Unknown	0 (0)	7 (8.8)	-	0.114
	Hispanic American countries	18 (90.0)	48 (60.0)	1	
Recommendations for people with cancer	Spain	1 (7.1)	15 (17.4)	0.27 (0.03–2.25)	0.203
	United States of America	0 (0)	11 (12.8)	-	0.109
	Others/Unknown	0 (0)	7 (8.1)	-	0.198
	Hispanic American countries	13 (92.9)	53 (61.6)	1	
Recommendations for people with obesity	Spain	0 (0)	16 (18.4)	-	0.054
	United States of America	0 (0)	11 (12.6)	-	0.109
	Others/Unknown	0 (0)	7 (8.0)	-	0.198
	Hispanic American countries	13 (100)	53 (60.9)	1	
Recommendations for healthcare workers	Spain	3 (16.7)	13 (15.9)	0.79 (0.19–3.12)	0.732
	United States of America	0 (0)	11 (13.4)	-	0.080
	Others/Unknown	0 (0)	7 (8.5)	-	0.159
	Hispanic American countries	15 (83.3)	51 (62.2)	1	
Recommendations for people with HIV ^d^	Spain	0 (0)	16 (17.6)	-	0.194
	United States of America	0 (0)	11 (12.1)	-	0.343
	Others/Unknown	0 (0)	7 (7.7)	-	0.586
	Hispanic American countries	9 (100)	57 (62.6)	1	
Recommendations for people with chronic kidney disease	Spain	0 (0)	16 (17.2)	-	0.336
	United States of America	0 (0)	11 (11.8)	-	0.584
	Others/Unknown	0 (0)	7 (7.5)	-	1.000
	Hispanic American countries	7 (100)	59 (63.5)	1	
Recommendations for people with diabetes mellitus	Spain	2 (9.5)	14 (17.7)	0.38 (0.08–1.85)	0.219
	United States of America	1 (4.8)	10 (12.7)	0.27 (0.03–2.24)	0.198
	Others/Unknown	0 (0)	7 (8.9)	-	0.114
	Hispanic American countries	18 (85.7)	48 (60.8)	1	
Recommendations for all people older than 6 months	Spain	1 (9.1)	15 (16.9)	0.42 (0.05–3.59)	0.421
	United States of America	1 (9.1)	10 (11.2)	0.63 (0.07–5.56)	0.679
	Others/Unknown	0 (0)	7 (7.9)	-	0.300
	Hispanic American countries	9 (81.8)	57 (64.0)	1	
Costs of the vaccine	Spain	1 (5.6)	15 (18.3)	0.19 (0.02–1.57)	0.093
	United States of America	0 (0)	11 (13.4)	-	0.058
	Others/Unknown	0 (0)	7 (8.5)	-	0.128
	Hispanic American countries	17 (94.4)	49 (59.8)	1	
Description of the dosage	Spain	0 (0)	16 (18.6)	-	0.054
	United States of America	1 (7.1)	10 (11.6)	0.41 (0.05–3.48)	0.402
	Others/Unknown	0 (0)	7 (8.1)	-	0.198
	Hispanic American countries	13 (92.9)	53 (61.6)	1	
Hoaxes and conspiracy theories	Spain	6 (31.6)	10 (12.4)	6.0 (1.61–22.34)	0.004
	United States of America	4 (21.0)	7 (8.6)	5.71 (1.29–25.29)	0.031
	Others/Unknown	3 (15.8)	4 (4.9)	7.5 (1.35–41.72)	0.036
	Hispanic American countries	6 (31.6)	60 (74.1)	1	

^a^ OR (95% CI): odds ratio (95% confidence interval). ^b^ Recommendations for pregnancy (from 13 weeks of gestation or in any trimester of pregnancy). ^c^ Recommendations for children of 6 months to 2/5/6/10 years. ^d^ HIV: human immunodeficiency virus.

## Data Availability

Data is contained within the article.
